# Plant Uptake of Persistent and Mobile Chemicals in Rocket (*Eruca sativa*)—A Greenhouse Study on Agricultural Wastewater Reuse

**DOI:** 10.1021/acs.est.5c02379

**Published:** 2025-04-28

**Authors:** Alina H. Seelig, Veikko Junghans, Thorsten Reemtsma, Daniel Zahn

**Affiliations:** † Department of Environmental Analytical Chemistry, Helmholtz-Centre for Environmental Research—UFZ, Permoserstrasse 15, 04318 Leipzig, Germany; ‡ Humboldt-Universität zu Berlin, Unter den Linden 6, 10099 Berlin, Germany; § Institute for Analytical Chemistry, University of Leipzig, Linnéstrasse 3, 04103 Leipzig, Germany

**Keywords:** PMT/vPvM, bioconcentration factor, bistriflimide, organic micropollutants, battery PFAS, ionic liquids, carbamazepine, horticulture

## Abstract

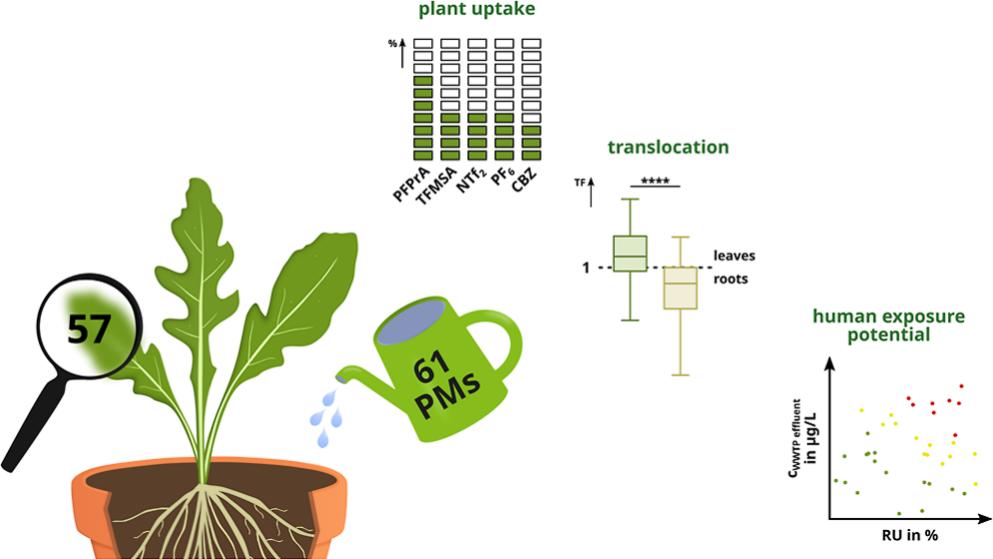

With increasing water stress, agricultural wastewater reuse is becoming more prevalent worldwide. In this context, persistent and mobile (PM) chemicals may be especially relevant, yet data on their uptake by plants are still scarce. This study investigates the uptake of 61 PM chemicals by and distribution in rocket (*Eruca sativa*) in pot experiments from spiked water and treated municipal wastewater. The relative uptake (RU; share of theoretical maximum uptake) into rocket exceeded 10% for 18 PM chemicals, among others, perfluoropropionic acid (72% RU), trifluoroacetic acid (67%), and tetrafluoroborate (40%). The median plant uptake (*p* ≤ 0.05) and translocation factor (*p* < 0.0001) were significantly higher for PM chemicals than reported for less polar chemicals into leafy greens from literature. Irrigation of rocket with reclaimed municipal wastewater resulted in the accumulation of 23 analytes. However, a toxicological threshold of concern approach showed that no critical exposure is reached for these compounds at normal consumption levels. Comparing wastewater treatment plant effluent concentrations with plant uptake data led to a prioritization of PM chemicals, which might be especially relevant for agricultural wastewater reuse and should be considered in risk assessment.

## Abstract 2

Many PM chemicals show an effective uptake by and translocation to edible parts in rocket, underpinning their relevance for risk assessment in agricultural wastewater reuse.

## Introduction

Groundwater levels and freshwater resources are declining, which can lead to increasing competition between agricultural irrigation, drinking water supply, and industrial use.^1^ Global freshwater withdrawal is predicted to increase by 10% by 2040 due to the increasing population and fast urbanization, which may further intensify competition for water resources.^2−4^ To counteract the water shortage, treated municipal wastewater may be used for nonpotable reuse by irrigation in agriculture or groundwater recharge.^5^ This reuse of wastewater is already commonplace in 24 countries dealing with high water stress by reusing >10% of reclaimed wastewater resources,^6^ and it will become prevalent in more countries if water shortages further increase.

Agriculture is responsible for 71% of the global freshwater withdrawal,^7^ and thus guidelines and regulations for agricultural wastewater reuse have been established by many countries and supranational organizations.^8−10^ The EU Regulation 2020/741,^11^ which came into force in June 2023, provides a harmonized framework for agricultural wastewater reuse across its member states and introduces a mandatory risk assessment approach to safeguard public health, the environment, and agricultural production. Persistent and mobile (PM) chemicals may deserve attention in this context, since wastewater treatment only leads to incomplete removal of many PM chemicals^12^ and may even result in their formation.^13^ Yet information on their uptake by and distribution in plants is still scarce.^14^ Two main aspects may contribute to this data scarcity: (i) PM chemicals have only come into focus of the scientific community and regulation in recent years,^15^ and (ii) analytical limitations often hamper their determination.^16^

Studies on the plant uptake of organic micropollutants are predominantly focused on pharmaceuticals and personal care products,^17−23^ long-chain perfluoroalkyl substances (PFAS),^24,25^ or benzotriazoles,^26^ many of which are less polar than the PM chemicals studied herein. Commonly used models disagree on whether highly polar chemicals are readily taken up into plant leaves or not,^27−31^ which may be a consequence of the data scarcity in this polarity range. At least in theory, polar chemicals should be translocated readily in plants if they can cross cell membranes quickly,^32^ highlighting the need for further investigation.

The uptake of chemicals by plants can be studied by various approaches that always represent a trade-off between realism and controlled conditions. While field experiments are performed under environmental conditions providing the most realistic results, they are time-consuming and difficult to replicate, and the limited control of experimental conditions renders it difficult to infer results beyond concentrations in different compartments.^33^ In addition, evapotranspiration rates, which are important for a more sophisticated interpretation, cannot be monitored. In contrast, pot experiments are performed under artificial but well-controlled conditions, which allow for carefully monitoring the exposure of individual plants to the test chemicals,^34^ thus facilitating a quantitative assessment of chemical uptake and distribution.

In this study, the plant uptake of 61 PM chemicals in rocket is investigated in pot experiments in a greenhouse. Concentrations in the roots and leaves as well as the water uptake of each individual pot were determined to assess the uptake and distribution of a wide range of PM chemicals in rocket and infer if trends based on physicochemical properties or structural similarities are apparent. Irrigation experiments with reclaimed wastewater were used to perform a risk assessment based on the threshold of toxicological concern (TTC). A comparison between plant uptake data reported herein and concentrations in reclaimed wastewater reported by Muschket et al.^35^ was used to prioritize PM chemicals with a high potential for human exposure through agricultural wastewater reuse, for which a sophisticated risk assessment seems appropriate.

## Materials and Methods

### Persistent and Mobile Chemicals

A total of 61 PM chemicals were analyzed in this study. The selection of the chemicals took place on the basis of a suspect screening by Neuwald et al.^36^ The list contains pharmaceuticals or industrial chemicals. Structural information as well as information regarding the analytical reference standards is provided in Table S1. Physicochemical properties of analytes ranged from −5.4 to 3.9 (log *D*_OW_), −0.6 to 4.3 (log *K*_OC_), and 6.4 × 10^–19^ to 3.5 × 10^–2^ (log *K*_AW_) and are provided in Table S2.

### Plant Cultivation

For plant cultivation, rocket (*Eruca sativa*) was selected as a representative for a wide variety of cultivated, fast-growing leafy vegetables. Since the harvested organs of leafy vegetables are mainly used fresh, e.g., in salads, a decomposition of PM chemicals in the plant leaves after harvesting through processing steps, like cooking, is not expected.

Rocket (var. “Speedy”) was cultivated in the greenhouse in Berlin-Dahlem (artificial light for 12 h d^–1^, mean temperature of 18 ± 2 °C, and mean relative humidity of 49 ± 13%) by using standard “Mitscherlich-pots” (max. vol. 6200 mL, diameter 20 cm, and height 21 cm), five plants per pot, each pot filled with 6500 g sieved (2 mm mesh size) and homogenized air-dry topsoil (Table 1). The resulting bulk density after initial filling was calculated to be 1.51 g cm^–3^.

**Table 1:** Physicochemical Properties of the Used Soil (*C*_org_: Organic Carbon, *C*_t_: Total Carbon, and *N*_t_: Total Nitrogen Content in wt. %). pH Values Measured 0.01 mol L^–1^ CaCl_2_ With a Soil Solution Ratio of 1:2.5. BS: Base Saturation-% of Ca^2+^, Mg^2+^, Na^+^, and K^+^ of the CEC_pot_)

texture class	textural fractions (%)			*C*_org_	*C*_t_	*N*_t_	pH CaCl_2_	CEC_pot_	BS
	sand	silt	clay	%				*C* mol kg^–1^	%
silty sand (Su3^a^)	61.0	36.2	2.8	0.20	0.30	0.02	5.7	2.09	62.5

a According to German soil classification.

All pots were regularly watered (maximal length of interval = 72 h) to a maximum field capacity of 80%. Leaching due to excessive irrigation of these pots was not registered. Rocket was watered by simulating drip irrigation, where leafy parts of the rocket did not have any contact with the irrigation water (in contrast to overhead or sprinkler irrigation). To estimate evaporation, unseeded pots were watered, using the same soil parameters, intervals, and irrigation thresholds (80%) as above. Additionally, no leaching was registered in unseeded pots. Transpiration was calculated by subtracting evaporation from overall water consumption, although this might be imprecise regarding the de facto evaporation from planted pots in later growth phases. To avoid unwanted growth depression, an initial fertilization [400 mg kg^–1^ d.w. Ca(NO_3_)_2_·4H_2_O, 150 (K), or 119 (P) mg kg^–1^ d.w. KH_2_PO_4_, 60 mg kg^–1^ d.w. MgSO_4_·7H_2_O] was given to all seeded pots.

### Exposure Experiments

Exposure experiments were performed with two types of water (spiked deionized tap water 10 μg L^–1^) and reclaimed wastewater with four replicates for each water type. The spiked concentration was selected based on previous works by Muschket et al.^35^ and Neuwald et al.,^12^ who reported up to 8 μg L^–1^ and 33 μg L^–1^, respectively, for individual PM chemicals in treated wastewater. The reclaimed wastewater originates from the municipal wastewater treatment plant (WWTP) in Berlin-Ruhleben (1,600,000 population equivalents), which contains a mechanical and biological treatment combined with UV-disinfection during the summer period.^37^ Here, samples were taken directly from the effluent every 2 weeks to monitor changes in its micropollutant composition and transported and stored in plastic containers.

Other irrigation water was stored in glass bottles. Spiked irrigation water was prepared using a mixture of PM chemicals described in the chapter “PM Chemicals”. The stock solution (10,000 μg L^–1^) was stored at 5 °C in dark conditions. Glass bottles with spiked water and containers with treated wastewater were stored under exclusion of light at ambient temperature. Also, samples of spiked water were collected and analyzed to detect changes in concentrations. All water samples were immediately deep-frozen after sampling. Irrigation was performed with separate watering cans made of glass for each water type to avoid cross-contamination.

After an initial seeding of ten seeds per pot, only five plants of rocket were cultivated to the harvest. Minimizing to five plants was carried out after 7 days of initial cultivation. Dry matter of the discarded plants was <5% of the total leaves’ harvest mass. Rocket irrigated with reclaimed wastewater was harvested multiple times, with the last edible yield being used for this study.

### Sample Preparation and Extraction

Rocket plant material (upper leaves, bottom near leaves, and roots) was harvested after 65 days of cultivation at BBCH stage 59, which was 7–10 days later than the typical harvest stage at BBCH stage 49 for leafy vegetables. To reduce contamination with soil, all harvested roots were washed with drinking water until they were free of soil.

Harvested plant material was transferred separately in paper bags and immediately dried at 30 °C in an oven for 48 h and subsequently homogenized with a ceramic ball mill (Retsch MM200) to a powder-like matrix. Homogenized plant material was transferred into glass bottles and stored in the dark at ambient temperature until further analysis.

All plastic materials used for sample preparation, as well as glass bottles for storing water and plant material, were rinsed twice with methanol before use. Glass bottles were heated for 4 h at 500 °C. Sample extraction was performed based on Riemenschneider et al.^38^ Therefore, 1 g of powdered plant material was weighed into centrifuge glass tubes, 3 mL of Milli-Q-water was added, and the mixture was soaked for 60 min. Then, 5 mL of methanol were added. After 20 min of ultrasonication and 30 min of shaking (∼200 rpm), the samples were centrifuged for 10 min (4 °C, 3000 g). The supernatant was collected and stored. The extraction steps were repeated with 5 mL of methanol/Milli-Q-water (1:1, v/v), and both supernatants were combined. An aliquot of this was centrifuged for 10 min (4 °C, 13,000 g), and the supernatant was transferred into glass HPLC vials. Some replicates were spiked with reference standards for the determination of recovery rates and matrix effects at 10 μg L^–1^.

Spiked water samples and WWTP effluent were centrifuged for 10 min (4 °C, 13,000 g), diluted 1:1 (v/v) with methanol, and transferred into glass HPLC vials. Replicates were spiked with a reference standard mix (1, 5, and 10 μg L^–1^) to calculate matrix effects for quantification.

### Instrumental Analysis

For sample analysis, supercritical fluid chromatography (Acquity UPC^2^ system) coupled to tandem mass spectrometry (Xevo TQ-XS), both from Waters (Milford, MA, USA), was used. The method was reported by Seelig et al.^39^ Measurements were performed by using two methods. A flow rate of 1.5 mL min^–1^ and a makeup flow rate of 0.3 mL min^–1^ were used for both methods using CO_2_ (eluent A) and 95% methanol, 5% Milli-Q-water and a buffer [eluent B; 10 mM ammonium formate (method A), 0.05% of a 25% ammonium hydroxide solution (method B)]. Method A was running for 17.2 min with a Waters Acquity UPC^2^ BEH (3.0 × 100 mm, 1.7 μm, 130 Å) column operated at 55 °C. A Waters Torus Diol column (3.0 × 150 mm, 1.7 μm, 130 Å) was used for method B with a run time of 9 min.

### Quantification of Target Analytes

Quantification was performed by using TargetLynx software from Waters (Milford, MA, USA). A 7-point matrix-matched calibration (0.01–10 μg L^–1^) was used to quantify the analytes in rocket roots and leaf extracts. The calibration was prepared in plant extracts irrigated with deionized tap water. All water samples were quantified with an external calibration (0.01–10 μg L^–1^, weighting of calibration regression line 1/*x*) and corrected for recovery rates and matrix effects through standard addition (1, 5, and 10 μg L^–1^). In general, calibration points with a deviation >25% were excluded. Method validation was performed for rocket leaf and root samples and is shown in Table S3. *R*^2^ was between 0.943 and 1.000. Method detection limits ranged from 1.7 ng g^–1^ to 5.6 μg g^–1^ (leaves) and from 5.2 ng g^–1^ to 6.2 μg g^–1^ (roots), and apparent recoveries ranged from 0.3 to 1.7 (leaves) and 0.2 to 1.8 (roots). Method validation for water samples was performed by Seelig et al.^39^

### Data Interpretation

For data interpretation, the relative uptake (RU; percentage of the theoretical maximum taken up) of the investigated PM chemicals by the plant was calculated. Based on eq 1, the product of the concentration in rocket material and the mass of fresh weight rocket material was divided by the product of the concentration in the irrigation water and the volume of water which was transferred into the plant
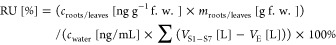
1where *V*_S1–S7_ is the volume of irrigation water for planted pots from batches 1 to 7, and *V*_E_ is the volume of irrigation water for unseeded (reference) pots. This calculation does not take diffusive uptake pathways for chemicals into roots into account.

The bioconcentration factor (BCF) was calculated based on eq 2
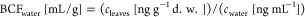
2

## Results and Discussion

### Concentrations in Roots and Leaves

After the cultivation period of 65 days, 57 of 61 PM chemicals spiked into the irrigation water were detected in the leaves of the rocket. The log *D*_OW_ of the test chemicals ranged from −5.4 to 3.9 with a mean value of −1.0; 41 of the chemicals were charged in the pH range of the water–soil–plant system (16 neutral analytes, 18 cations, and 23 anions). Mean concentrations of the 57 chemicals in the plant material ranged from 0.1 to 1106 ng g^–1^ f.w. (mean relative standard deviation (sd) of the four replicates of 22%) in leaves and 0.2 to 96 ng g^–1^ f.w. (mean relative sd of 50%) in roots (Table S4), showing less accumulation in the roots compared to leaves. Saccharine, perfluoropropanesulfonic acid (PFPrS), 4,4′-methylenedianiline, and 3-(acryloyloxy)-1-propanesulfonic acid were not detected in plant material. Additionally, 1-(2-hydroxyethyl)-2,2,6,6-tetramethyl-4-piperidinol, cumenesulfonic acid, diclofenac (DCF), and 3,5-bis(methoxycarbonyl)benzenesulfonic acid were not detected in rocket roots.

### Plant Uptake of Persistent and Mobile Chemicals in Rocket

**Figure 1:**
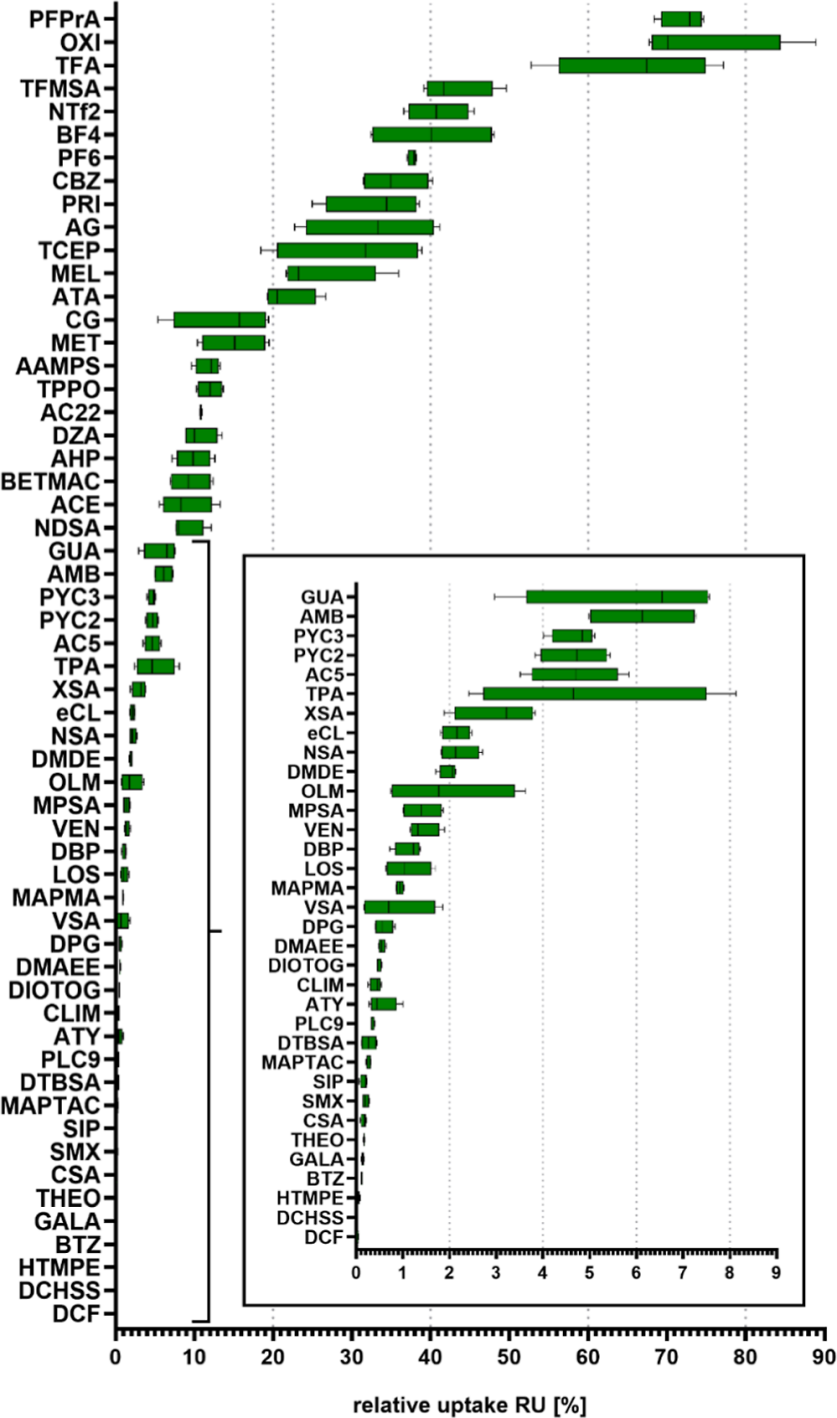
RU for all detected analytes in rocket. Crops were irrigated with freshwater spiked with 10 μg L^–1^ of reference substances. Four pots were grown and analyzed individually.

**Figure 2:**
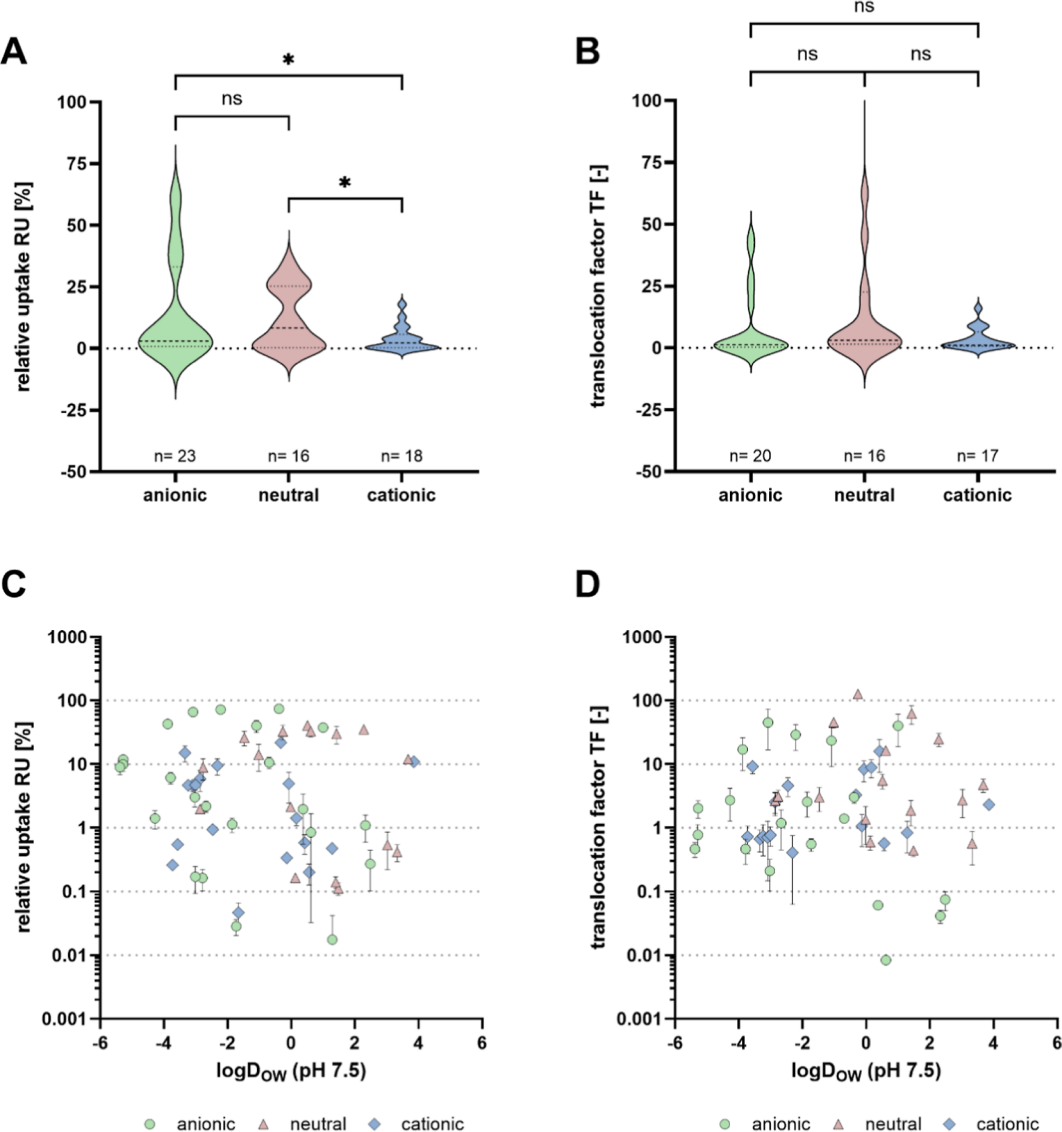
(a,b) Violin plots of the RU (a) and the translocation factor (TF) (b) of detected PM chemicals of different charge states at pH 7.5. ns: not significant. *: *p*-value of *t*-test ≤0.05. (c,d) Comparison between RU (c) or TF (d) and the log *D*_OW_ (at pH 7.5). Anionic compounds in green, neutral analytes in red, and cationic substances in blue. Irrigation with 10 μg L^–1^ of reference substances. Four replicates were grown individually.

The theoretical maximum uptake of a chemical by a plant is determined by the amount of water taken up by the plant over the growth period (transpiration rate of the four replicates: 56–58% with a relative sd of 1.4%) and the concentration of the chemical in the irrigation water. The RU (in %) actually detected in the plant (eq 1) must not exceed this theoretical maximum since a chemical may be partially adsorbed to soil, from bound residues, or may undergo transformation in the soil or plants^40−42^ or be excreted from the plant^43^ during the growth period. The median RU for the investigated PM chemicals ranged from 0.01% (DCF) to 73% [perfluoropropionic acid (PFPrA)] in rocket (Figure 1), thus spanning 4 orders of magnitude (Figure 2c).

Accumulation of chemicals in plant leaves is a multistep process beginning with the uptake of chemicals by the roots, followed by a translocation with the xylem toward the leafy greens, and ending with the evaporation of water and volatile chemicals from those leaves. The majority of the investigated chemicals (88%) are not volatile (log *K*_AW_ value <10^–6^; Table S2). However, volatilization may be relevant for 1-adamantanamine, bistriflimide (NTf2), PFPrA, PFPrS, trifluoroacetic acid (TFA), and tripropylamine, three of which show among the highest RU values in this study and thus suggest a low influence of volatilization on the data set. The extents of accumulation and transport may be influenced by each of these steps. In general, plant uptake of organic micropollutants may be affected by various physicochemical properties, e.g., molecular mass, log *D*_OW_, p*K*_a_, pH or ionic strength.^44,45^ Only three analytes exceed a molecular weight of 400 g mol^–1^ where size exclusion by the Casparian strip^46^ becomes relevant. Of these analytes, only diatrizoic acid (DZA) showed significant plant uptake [DZA, mean RU 10%; olmesartan, mean RU 1.8%; and losartan, mean RU 1.0%]. The mean RU was 15% for neutral chemicals (*n* = 16; log *D*_OW_ range −2.9–3.7), 4.8% for cations (*n* = 18; log *D*_OW_ range −3.7–3.9), and 17% for anions (*n* = 23; log *D*_OW_ range −5.4–2.5, all at pH 7.5). While no significant difference was observed between anions and neutral chemicals, the uptake of cations was significantly lower (*p* ≤ 0.05 compared to neutral chemicals and anions; Figure 2a), which may be explained by their higher sorption tendency to soil.

Interestingly, all seven chemicals with the highest plant uptake in this study were anions, reaching up to 72% (PFPrA) of RU (Figure 1). This indicates that some of these low molecular-weight anions may readily overcome the charged cell membranes, potentially as neutral salts via ion channels or water pores, as it is described for minerals^47,48^ or via an apoplastic uptake pathway. Furthermore, this might result from fewer adsorption processes between anions and the cell membranes in the inner root space compared to neutral compounds or cations. Along the pH gradient (pH 5.5–7.5)^49−51^ only 4,4′-(oxidi-2,1-ethanediyl) dimorpholine underwent a change in its dominant charge state (positive at pH 5.5; neutral at pH 7.5). Within this data set, no clear correlation between individual physicochemical parameters and plant uptake was observed (Figures 2c and S1), which may be a consequence of loss processes like transformation^40,41^ or bound residue formation^42^ after the plant uptake of the chemicals. Furthermore, it might be possible that adsorption and thus lipophilicity may no longer be relevant parameters below a certain threshold.

### Bioconcentration Factors for Persistent and Mobile Chemicals in Rocket

**Figure 3:**
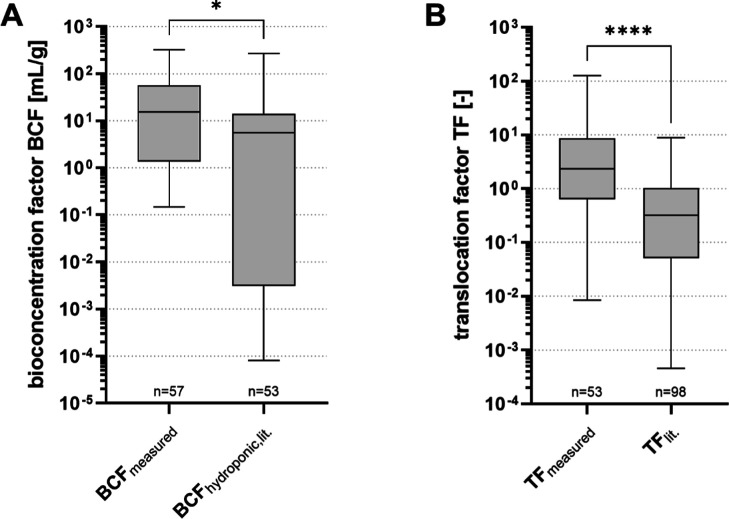
(a) Comparison of mean BCF_water_ determined in this study with BCF_water_ values reported for other compounds in hydroponic systems from literature data.^52−56^ *: *p*-value of *t*-test ≤0.05. (b) Comparison of mean TF to literature data from.^52,54,55,57−60^ ****: *p*-value of *t*-test <0.0001. Literature studies either reported BCF_water_ or TF values directly or provided sufficient information for their calculation. Only results for the plant uptake of leafy greens (e.g., lettuce) were used for comparison.

While the RU is well suited for reporting the accumulation of chemicals in plants since it accounts for their uptake of water and thus enhances comparability across studies, the amount of water taken up by the plants is not regularly reported. For that reason, the BCF is the more commonly reported parameter to quantify the enrichment of chemicals from the surrounding media to the plant and thus enables a much broader data basis for a comparison with the literature. Since most PM chemicals adsorb only poorly to the soil matrix, their soil concentration predominantly depends on the soil moisture when sampling took place instead of sorption processes, resulting in lower soil concentrations and thus overestimations of the BCF compared to less polar chemicals that are more efficiently retained by the soil. Consequently, BCFs were calculated based on irrigation water concentrations (eq 2, named BCF_water_ in the following) and compared to data reported for studies using hydroponic systems with similar cultivation times (21–55 days) and plant species (rocket, lettuce, or spinach) to reduce the impact of experimental variations. Figure 3a shows a significant difference (*p* ≤ 0.05) between the BCF_water_ values of the 57 analytes studied herein [median BCF_water_ 15; interquartile range (IQR) 1.4–53] compared to BCF_water_s of 53 less polar chemicals (median BCF_water_ 5.6; IQR 3.2 × 10^–3^–14) from literature studies,^52−56^ thus indicating that PM chemicals might be taken up more effectively by the rocket than many nonpolar chemicals.

Carbamazepine (CBZ) has been widely studied in various plant types (e.g., tomatoes, lettuce, or carrots^18,19,21^), where it shows generally high uptake but can vary between plant type and experiment. The CBZs BCF_water_ observed herein (mean BCF_water_ 153) is roughly twice as high as reported in the hydroponic systems used for comparison (BCF_water_ 68–92^52,56^), which might be a consequence of experimental variations (e.g., cultivation period).

Among the investigated chemicals with high uptake potentials are four ultrashort-chain PFAS, namely, PFPrA (mean BCF_water_ 312, Figure S2), TFA (294), trifluoromethanesulfonic acid (TFMSA; 188), and NTf2 (171). While the plant uptake of PFAS with a chain length > C4 has been already widely studied previously,^54,61^ this does not apply to ultrashort-chain PFAS in the context of plant uptake. The results for PFPrA and TFA are in line with the results of Dal Ferro et al.,^54^ who investigated the plant uptake of PFAS with a chain length > C4 and observed BCF_water_s increasing with decreasing chain lengths, reaching 240 in lettuce and 66.3 in spinach for PFBA, the shortest chain PFAS tested in that study. The results shown herein indicate this trend holds true for even shorter chain lengths of perfluoroalkyl carboxylic acids (PFCAs), which is in line with a study investigating PFAS in wheat plants,^48^ and may be extrapolated to PFAS of other classes like the sulfonylimide NTf2. NTf2 was first reported in the environment in 2021^36^ and is used as an electrolyte in lithium-ion batteries.^62^ While it is often detected in low concentrations,^63^ its presence is expected to increase with a shift toward a green energy infrastructure.^64^ Interestingly, a high BCF_water_ was observed for TFMSA (188), while perfluoroalkylsulfonic acids (PFSAs) are generally reported with much lower BCF_water_s than the corresponding PFCAs (e.g., PFPeA 58 and PFBS 6.2 in lettuce^54^). Even slightly longer chain PFSAs like PFPrS showed no bioconcentration in this study, and thus this strong PFSA uptake might be unique to TFMSA.

With tetrafluoroborate (BF4; mean BCF_water_ 175) and hexafluorophosphate (PF6; mean BCF_water_ 166), two inorganic anions widely used in ionic liquids showed high BCF_water_ values. These novel micropollutants seem widely spread in aquatic environments, and BF4 was reported with maximum concentrations in the μg L^–1^ range in surface waters,^36^ thus their human exposure through crop consumption may be likely even when surface water is used for irrigation.

### Translocation of Persistent and Mobile Chemicals within Rocket

After the root cortex is passed, chemicals can be transported to the above-ground part of the plant. The efficacy of this transport can be expressed by the ratio of concentrations in leaves and roots (TF; Figure 2d). Here, loss processes could not be considered when calculating the TF. TF values ranged from 8.4 × 10^–3^ (valsartanic acid) to 126 (acetoguanamine) with a median value of 2.3 and an IQR of 0.7–8.3 (Figure 3b). Literature studies^52,54,55,57−60^ analyzing 48 less polar compounds (mean log *D*_OW_ 1.5 at pH 7.5) in lettuce and spinach reported TF values with an IQR (5.3 × 10^–2^–1.0) and median (0.3) significantly (*p* < 0.0001) lower than for the PM chemicals tested herein, indicating a more pronounced transport for these more polar chemicals (mean log *D*_OW_ −1 at pH 7.5). This is consistent with the overall theory that highly polar substances are readily transferred via the xylem into the stem and leaves of the plant^65^ and may be the main reason for the high observed BCF_water_ of PM chemicals.

While some models for uptake and enrichment predict poor enrichment of polar chemicals in edible parts (e.g., the bell-shaped model with an optimum around log *K*_OW_ of 2 by Briggs et al. and others^27−31^), others predict an increasing enrichment at lower log *K*_OW_ (e.g., the sigmoidal model by Dettenmaier et al.^14^). The large data set for plant uptake and translocation generated herein contains 39 chemicals with log *K*_OW_ < 0, which show a large variability in translocation (mean TFs 8.4 × 10^–3^–126), but no clear trend with log *K*_OW_ or charge state was evident (Figure 2b–d). Shorter growth periods of rockets may also reduce accumulation and translocation.

### Threshold of Toxicological Concern Assessment for Wastewater-Irrigated Rocket

The reclaimed municipal wastewater of WWTP Ruhleben contained 39 of the 61 PM chemicals analyzed herein above their limit of quantification. Irrigating rocket plants with WWTP effluent resulted in the detection of 23 PM chemicals in concentrations between 0.2 (1,3-di-*o*-tolylguanidine) and 115 ng g^–1^ (PF6) fresh weight (f.w.) in rocket leaves (Figure S3). Here, seven analytes were detected above 10 ng g^–1^ f.w., namely, PF6 (115 ng g^–1^), melamine (MEL, 45 ng g^–1^), vincubine (31 ng g^–1^), BF4 (16 ng g^–1^), ε-caprolactam (15 ng g^–1^), guanylurea (GUA, 13 ng g^–1^), and CBZ (11 ng g^–1^). Concentrations in leaves irrigated with a wastewater/deionized tap water mix (50:50, v/v) resulted in leaf concentration ratios between both irrigations of 1.6 ± 0.9 (Figure S4), thus demonstrating that small concentration deviations in wastewater do not influence the uptake process strongly. The small deviation from the expected ratio of 2 is partially caused by some of the PM chemicals being present in deionized tap water.

Since in vivo toxicity data are not available for 56% of the 23 PM chemicals detected in wastewater-irrigated rocket in the U.S. EPA’s CompTox database,^66^ the TTC approach was used to define exposure levels below which the risk for human health is assumed negligible and put them in relation to exposure through consumption of wastewater-irrigated rocket. TTC values are calculated based on Cramer et al.,^67^ who classified chemicals into three groups (Cramer classes I–III) with threshold doses of 30, 9, and 1.5 μg per kg bodyweight (b.w.) and day, respectively.^68^ These groups are based on the fifth percentile of the no observed effect levels of structurally similar chemicals. It has to be noted that PF6, BF4, gabapentin lactam, and ethyltrimethylammonium could not be assigned a Cramer Class by using Toxtree software.^69^ From these TTC values and the concentrations detected in rocket leaves irrigated with reclaimed wastewater, the maximum daily consumption for an average adult (assuming 70 kg b.w.) was calculated. The lowest permitted consumption level was calculated for MEL (Cramer class III) with 2.3 kg per person (Table S5). Riemenschneider et al.^21^ reported similar values with a maximum daily consumption for CBZ of 18 kg per person and a day of rocket. These values exceed the daily consumption of all vegetables recommended by the German Nutrition Society (∼400 g) significantly. However, vulnerable groups such as older or sick people and children may show detrimental effects at lower exposure levels; other exposure routes (e.g., drinking water consumption) can contribute to human exposure to PM chemicals. The Cramer classification is based on the assumption that chemicals with a similar structure result in a similar toxicity range, which may not necessarily be true, and not all chemicals assessed could be assigned a Cramer class.

### Human Exposure Potential through Agricultural Wastewater Reuse

**Figure 4:**
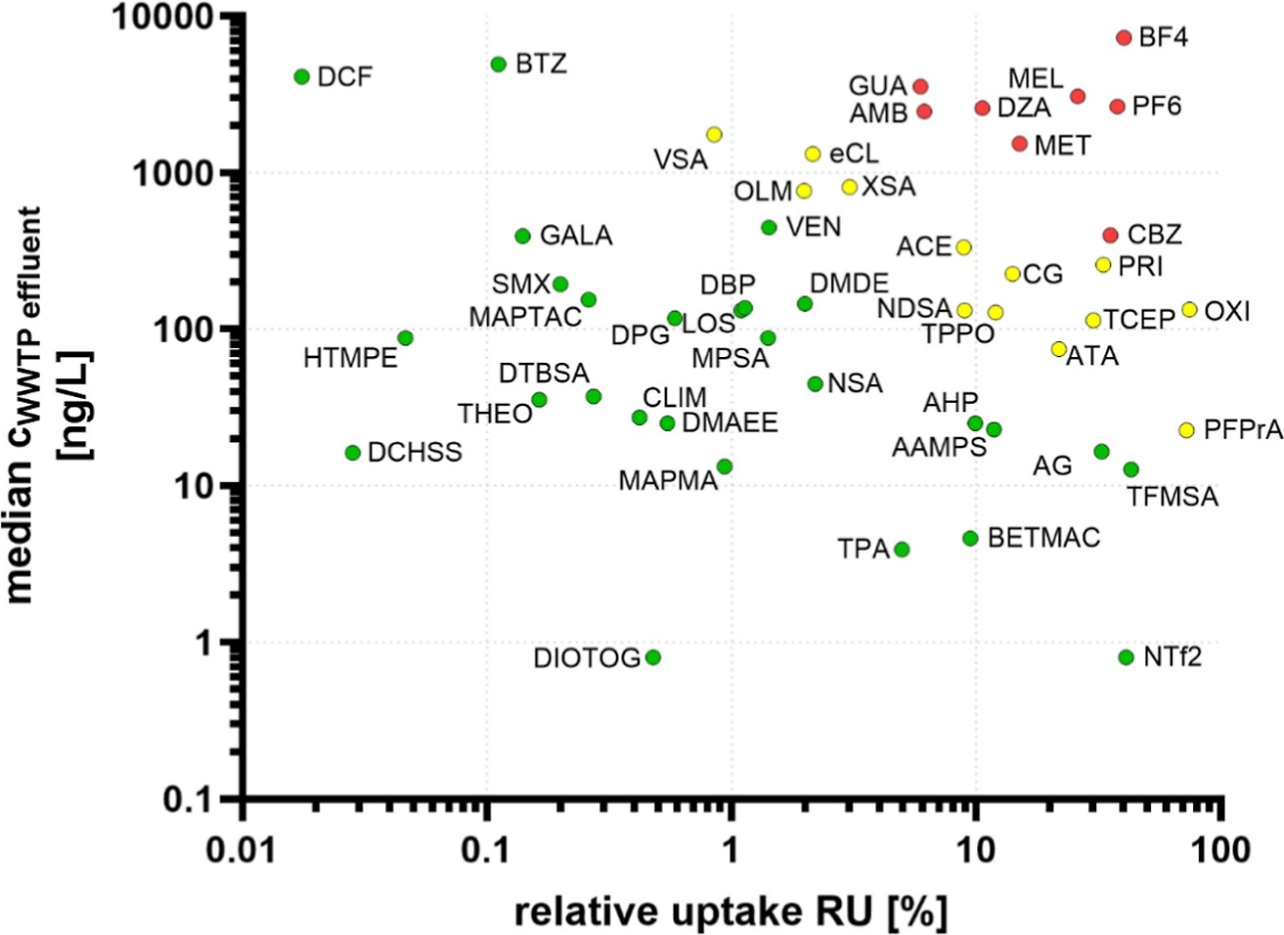
Double logarithmic scatter plot of median concentrations in WWTP effluent (*n* = 47) reported by Muschket et al.^35^ versus mean RU of the spike experiment (10 μg L^–1^). Categorization into three groups was performed based on their exposure potential [<1000 (green), 1000–10,000 (yellow), and >10,000 (red)].

Toxicity data is still scarce for many PM chemicals, and thus the human exposure potential of a chemical during agricultural wastewater reuse may be helpful to prioritize chemicals for which such data need to be generated as a part of a more sophisticated risk assessment. Chemicals that combine efficient uptake into plants (here expressed by the RU) with elevated concentrations in WWTP effluent seem especially relevant. By combining the RU values generated herein with median WWTP effluent concentrations (6 German WWTPs, *n* = 47) reported by Muschket et al.^35^ (Figure 4), the analytes were categorized into three groups based on their product of the two parameters (<1000, 1000–10,000, and >10,000), called “exposure potential” in the following.

The group with the highest exposure potential (*c* (ng L^–1^) × RU (%) > 10,000 [ng % L^–1^]) comprised eight chemicals, namely CBZ (one of the most often reported chemicals in plant uptake studies), 4-amino-3-methylbenzenesulfonic acid (AMB), GUA, metformin (MET), DZA, MEL, PF6, and BF4. Interestingly, the ionic liquid anions PF6 and BF4, the two chemicals with the highest exposure potential, were also among the chemicals which could not be assigned a Cramer class due to their pronounced structural difference from the chemicals for which the Cramer classification was established.^67^ The ultrashort-chain PFAS PFPrA, TFMSA, and NTf2 were among the chemicals with the highest RU but were detected in comparatively low concentrations in WWTP effluents (22.6, 12.7, and 0.8 ng L^–1^), resulting in a reduced human exposure potential. However, wastewater concentrations of chemicals can differ greatly between WWTPs or regions, especially if industrial wastewater contributes significantly to the influent, and thus, individual chemicals may show higher concentrations in specific effluents. Also, other aspects, such as fluctuating RU capacities for different plant types, can influence the exposure potential as well.

### Environmental Implications

An improved understanding of which wastewater-borne chemicals are readily taken up by plants is essential to inform risk assessment approaches to safeguard public health in agricultural wastewater reuse scenarios. Plant uptake has been widely studied for a variety of chemical classes, such as pharmaceuticals and pesticides, yet few of these chemicals are mobile and persistent, resulting in data scarcity for highly polar chemicals. In this study, investigating the uptake of 61 polar chemicals by rocket, 93% of these chemicals were detected in the edible part of the plant with median RUs ranging from 0.01% (DCF) up to 73% (PFPrA) when irrigated with spiked water (10 μg L^–1^). While BCFs (*p* ≤ 0.05) and TFs (*p* < 0.0001) were significantly higher than literature data for less polar chemicals, no clear correlation with the log *D*_OW_, and only a weak impact of the charge state could be observed within this data set. This supports the hypothesis that log *D*_OW_/log *K*_OW_ is not well-suited to predict the uptake and translocation of highly polar chemicals.^70^ The complex multistep process of uptake and translocation as well as possible loss processes (e.g., degradation, volatilization) which are not fully understood so far cannot be adequatly described by a single parameter. Additionally, there is a high uncertainty in the often-predicted log *D*_OW_/log *K*_OW_ values and an ongoing discussion to what extent log *D*_OW_/log *K*_OW_ accurately reflect a chemical’s polarity.^71,72^ Thus, more experimental data seems essential for prioritization of chemicals and further refinement of models in this polarity range. Since volatilization seems unlikely for many of the studied PM chemicals, a better understanding of the other loss processes, like transformation and formation of bound residues, may be essential to enhance predictive capabilities. Additionally, other types of vegetables as well as other soil types might show different effects in terms of accumulation and translocation of PM substances and should be examined in further research.

Many ultrashort-chain PFAS were among the chemicals with the strongest enrichment in rocket. While a pronounced accumulation in plants is well-known for PFCAs, this study demonstrates that PFAS of other classes, such as TFMSA and NTf2, may also accumulate strongly in plants. Especially, the pronounced enrichment of TFMSA is remarkable and contrasts with the generally low bioaccumulation in plants reported for longer-chain PFSAs.^54^ However, a high uptake into plants does not necessarily lead to high crop concentrations after irrigation with reclaimed wastewater, and thus, the accumulation in plants studied herein was compared to concentrations in WWTP effluents to identify chemicals with a high potential for human exposure. Among these, the inorganic ionic liquid anions PF6 and BF4 showed the highest human exposure potential through crop consumption since they combine high wastewater concentrations with an effective transfer into rocket. If this high uptake potential for highly polar chemicals is confirmed for a wider set of crops, then a more sophisticated risk assessment seems necessary. Such a risk assessment is currently hampered by the scarcity of experimental toxicology data. The extensive U.S. EPA Tox database^66^ reported in vivo toxicity data for only half of the 23 chemicals detected in rocket after irrigation with reclaimed municipal wastewater. Consequently, PM chemicals should be evaluated critically in the context of agricultural wastewater reuse, as some seem to combine a low potential for removal during water treatment with a high potential for plant uptake. The data reported herein may serve as a starting point to prioritize in such a process.

## Supporting Information Available

Analyzed chemicals and methods, concentration data of PM chemicals for spike and wastewater experiments, principle component analysis for the influence of parameters on the RU, PFAS BCFs, and edible amounts according to TTC evaluation (PDF)
