# Supplementary material for: Plant Uptake of Persistent and Mobile Chemicals in Rocket (Eruca sativa)—A Greenhouse Study on Agricultural Wastewater Reuse
Source: Environ Sci Technol. 2025 Apr 28;59(18):9265–74. doi: 10.1021/acs.est.5c02379 (PMC12080245; doi:10.1021/acs.est.5c02379)
Supplement: Supplementary file 1 — es5c02379_si_001.pdf [file es5c02379_si_001.pdf]

Supporting Information

Plant Uptake of Persistent and Mobile Chemicals in rocket (*Eruca sativa*) – A Greenhouse Study on Agricultural Wastewater Reuse

Environmental Science & Technology

Alina H. Seelig[1], Veikko Junghans[2], Thorsten Reemtsma[1,3], Daniel Zahn[1*]

[1] Department of Environmental Analytical Chemistry, Helmholtz-Centre for Environmental Research – UFZ, Permoserstrasse 15, 04318 Leipzig, Germany

[2] Humboldt-Universität zu Berlin, Unter den Linden 6, 10099 Berlin, Germany

[3] Institute for Analytical Chemistry, University of Leipzig, Linnéstrasse 3, 04103 Leipzig, Germany

* Corresponding author: Daniel Zahn, daniel.zahn@ufz.de

Summary: 12 pages, 4 figures, 5 tables.

Table of contents

Material and Methods

Table S1. List of analyzed PM chemicals with structural information and suppliers. S2

Table S2. List of analyzed PM chemicals with physico-chemical properties. S4

Table S3. Method validation parameters for plant material and soil. S6

Results and Discussion

Table S4. Concentration of PM chemicals in rocket leaves and roots in the spike experiment. S8

Figure S1. Principal component analysis of %RU values and physico-chemical properties. S9

Figure S2. Bioconcentration factors of ultrashort-chain PFAS. S9

Figure S3. Concentrations of detected PM chemicals in WWTP effluent and plant leaves. S10

Figure S4. Dependence on concentration of irrigation water. S11

Table S5. Maximum edible amount of rocket without exceeding the TTC. S11

References S12

Material & Methods

Table S1. List of analyzed PM chemicals with structural information and suppliers.

Name	Abbreviation	CAS	Monoisotopic mass in g/mol	Category a)	Supplier	Purity in %
2-acrylamido-2-methylpropanesulphonic acid	AAMPS	15214-89-8	207.1	industrial chemicals	Sigma-Aldrich	99
N-decyl-N,N-dimethyl-1-decanaminium	AC22	20256-56-8	326.4	pharmaceuticals	Sigma-Aldrich	98
ethyltrimethylammonium	AC5	15302-88-2	88.1	not specified	trc	-
acesulfame	ACE	33665-90-6	163.0	pharmaceuticals	Sigma-Aldrich	> 99
acetoguanamine	AG	542-02-9	125.1	industrial chemicals	Sigma-Aldrich	98
3-(allyloxy)-2-hydroxy-1-propanesulfonic acid	AHP	52556-42-0	195.0	industrial chemicals	Sigma-Aldrich	39-42<br>40% wt in H_2O
4-amino-3-methylbenzenesulfonic acid	AMB	63450-43-1	186.0	industrial chemicals	TCI	> 98
3-(acryloyloxy)-1-propanesulfonic acid	AOPSO3	31098-20-1	193.0	industrial chemicals	fluorochem	99
1-adamantanamine	ATA	768-94-5	151.1	both	fluorochem	97
ametryn	ATY	834-12-8	227.1	industrial chemicals	Sigma-Aldrich	98.5
N,N,N-trimethyl(phenyl)methaneaminium	BETMAC	14800-24-9	150.1	not specified	Sigma-Aldrich	97
tetrafluoroborate	BF4	14874-70-5	88.0	not specified	abcr	95
1H-benzotriazole	BTZ	95-14-7	119.0	industrial chemicals	Dr. Ehrenstorfer	99.5
carbamazepine	CBZ	298-46-4	236.1	pharmaceuticals	Sigma-Aldrich	≥ 98
1-cyanoguanidine	CG	461-58-5	84.0	industrial chemicals	Acros	99.9
climbazole	CLIM	38083-17-9	292.1	both	HPC	99.8
p-cumenesulfonic acid	CSA	16066-35-6	200.1	not specified	BLDpharm	98
dibutylphosphate	DBP	107-66-4	210.1	industrial chemicals	J&K	97
diclofenac	DCF	15307-86-5	295.0	both	Sigma-Aldrich	-
dicyclohexyl sulfosuccinate	DCHSS	137361-04-7	362.1	not specified	Sigma-Aldrich	> 98
1,3-di-o-tolylguanidine	DIOTOG	97-39-2	239.1	industrial chemicals	Sigma-Aldrich	99
2-[2-(dimethylamino)ethoxy]ethanol	DMAEE	1704-62-7	133.1	industrial chemicals	Sigma-Aldrich	98
dimorpholinodiethyl ether	DMDE	6425-39-4	244.2	industrial chemicals	Merck	99.2
1,3-diphenylguanidine	DPG	102-06-7	211.1	both	Sigma-Aldrich	97
3,5-ditertbutyl salicylic acid	DTBSA	19715-19-6	250.2	industrial chemicals	Sigma-Aldrich	≥ 98
diatrizoic acid	DZA	117-96-4	613.8	both	Sigma-Aldrich	99
ε-caprolactam	εCL	105-60-2	113.1	industrial chemicals	fluorochem	98
gabapentin lactam	GALA	64744-50-9	153.1	industrial chemicals	Acros Organics	98
guanylurea	GUA	141-83-3	102.1	not specified	abcr	98
1-(2-hydroxyethyl)-2,2,6,6-tetramethyl-4-piperidinol	HTMPE	52722-86-8	201.2	industrial chemicals	Ambeed	98

losartan	LOS	114798-26-4	422.2	both	fluorochem	95
N-[3-(dimethylamino)propyl]-2-methylacrylamide	MAPMA	5205-93-6	170.1	industrial chemicals	abcr	50 aq
3-(methacryloylamino)-N,N,N-trimethyl-1-propanaminium chloride	MAPTAC	51410-72-1	185.2	industrial chemicals	Sigma-Aldrich	≥ 97
4,4'-methylenedianiline	MDA	101-77-9	198.1	industrial chemicals	Sigma-Aldrich	> 99
melamine	MEL	108-78-1	126.1	industrial chemicals	MP	≥ 98
metformin	MET	657-24-9	129.1	pharmaceuticals	Sigma-Aldrich	98
2-methyl-2-propene-1-sulfonic acid	MPSA	1561-92-8	135.0	industrial chemicals	abcr	98
1,5-naphthalenedisulfonic acid	NDSA	81-04-9	288.0	industrial chemicals	fluorochem	-
naphthalene-1-sulfonic acid	NSA	85-47-2	208.0	industrial chemicals	abcr	97
bistriflimide	NTf2	82113-65-3	280.9	not specified	Sigma-Aldrich	> 98
olmesartan	OLM	144689-24-7	446.2	both	Sigma-Aldrich	> 98
oxipurinol	OXI	2465-59-0	152.0	both	Sigma-Aldrich	> 98.5
hexafluorophosphate	PF6	16919-18-9	145.0	industrial chemicals	Wellington	98
pentafluoropropionic acid	PFPrA	422-64-0	164.0	industrial chemicals	Wellington	98
pentafluoropropanesulfonic acid	PFPrS	423-41-6	249.9	not specified	Combi-Blocks	95
1-methyl-1-octylpyrrolidinium	PLC9	927021-43-0	198.2	industrial chemicals	Fluka	-
primidone	PRI	125-33-7	218.1	pharmaceuticals	fluorochem	99
1-ethylpyridinium	PYC2	15302-96-2	108.1	not specified	trc	-
1-propylpyridinium	PYC3	45705-28-0	122.1	not specified	Sigma-Aldrich	99.9
saccharin	SAC	81-07-2	183.0	both	Alfa Aesar	98
3,5-bis(methoxycarbonyl)benzenesulfonic acid	SIP	138-25-0	274.0	industrial chemicals	Fluka	-
sulfamethoxazole	SMX	723-46-6	253.1	pharmaceuticals	fluorochem	97
tris(2-chloroethyl) phosphate	TCEP	115-96-8	284.0	industrial chemicals	Biosolve	-
trifluoroacetic acid	TFA	76-05-1	114.0	industrial chemicals	Sigma-Aldrich	98
trifluoromethanesulfonic acid	TFMSA	1493-13-6	150.0	industrial chemicals	Sigma-Aldrich	-
theophylline	THEO	58-55-9	180.1	both	Sigma-Aldrich	> 98
tripropylamine	TPA	102-69-2	143.2	industrial chemicals	fluorochem	99
triphenylphosphine oxide	TPPO	791-28-6	278.1	industrial chemicals	Sigma-Aldrich	≥ 98
venlafaxine	VEN	93413-69-5	277.2	both	Campro	-
valsartanic acid	VSA	164265-78-5	266.1	not specified	Sigma-Aldrich	> 91
3,4-xylenesulfonic acid	XSA	1300-72-7	186.0	not specified	Sigma-Aldrich	99

[a)] industrial chemical – when provided by ECHA[1], pharmaceutical – when listed in the "Model List of Essential Medicines" of the WHO or in DrugBank[2]

Table S2. List of analyzed PM chemicals with physico-chemical properties.

Abbreviation	logD (pH 5.5) [b)]	logD (pH 7.4) [b)]	log K_{OC} [b)]	ionic state (pH 7.4) [b)]			pK_A [b)]	log K_{AW} [c)] (25 °C)
				+	0	-		
AAMPS	-5.28	-5.40	0.41			1	-1.9<br>1.2<br>14.2	2.12×10^{-13}
AC22	3.85	3.85	2.74	1			-	1.77×10^{-9}
AC5	-3.10	-3.10	-0.23	1			-	1.43×10^{-11}
ACE	-2.77	-2.77	1.20		1		-0.4<br>9.0	3.94×10^{-7}
AG	-0.27	-0.18	1.16		1		-1.2<br>4.4	2.41×10^{-8}
AHP	-5.29	-5.29	0.64			1	1.1<br>14.4	5.10×10^{-13}
AMB	-3.79	-3.80	1.35			1	0.3<br>3.3	4.01×10^{-11}
AOPSO3	-4.61	-4.74	0.95			1	1.4	1.14×10^{-9}
ATA	-0.33	-0.37	2.58	1			10.5	3.40×10^{-4}
ATY	3.02	3.04	3.06		1		-1.5<br>4.1	2.80×10^{-7}
BETMAC	-2.32	-2.32	0.36	1			-	8.69×10^{-13}
BF4	-	-	-			1	-	7.89×10^{-6}
BTZ	1.48	1.44	2.00		0.9	0.1	1.6<br>8.5	6.00×10^{-6}
CBZ	2.28	2.28	2.83		1		0.1<br>14.3	4.42×10^{-9}
CG	-1.03	-1.03	0.75		1		-0.9<br>13.0	9.20×10^{-9}
CLIM	2.94	3.32	3.15	0.1	0.9		6.6	1.17×10^{-7}
CSA	-2.79	-2.79	2.36			1	0.7	2.00×10^{-7}
DBP	-1.86	-1.99	2.57			1	1.5	1.74×10^{-7}
DCF	3.14	1.37	3.58			1	4.4	1.94×10^{-10}
DCHSS	-1.73	-1.74	3.02			1	-3.7	1.28×10^{-11}
DIOTOG	1.28	2.24	3.43	1			10.1	3.54×10^{-10}
DMAEE	-3.57	-2.06	0.57	1			8.7<br>14.9	1.13×10^{-9}
DMDE	-2.87	-0.81	0.78	0.1	0.9		5.5<br>6.5	7.17×10^{-13}
DPG	0.42	1.54	2.93	1			10.1	2.91×10^{-10}
DTBSA	2.48	2.14	4.34			1	3.0<br>13.3	3.87×10^{-6}
DZA	-0.70	-1.00	1.62			1	1.4<br>11.0<br>12.1	1.15×10^{-16}
εCL	-0.02	-0.02	1.20		1		-0.3<br>15.1	1.04×10^{-6}
GALA	1.40	1.40	1.72		1		-0.3<br>15.1	1.07×10^{-6}
GUA	-2.87	-1.82	0.35	0.7	0.3		7.8	1.01×10^{-13}
HTMPE	-1.66	-0.97	2.07	0.9	0.1		8.5<br>14.7<br>15.5	8.49×10^{-9}
LOS	2.33	1.57	3.32			1	3.3<br>4.1<br>15.5	1.78×10^{-14}
MAPMA	-2.47	-1.22	1.49	1			-1.5<br>9.2<br>14.8	7.25×10^{-10}

MAPTAC	-3.73	-3.73	-0.64	1			-1.5<br>13.6	5.23×10^{-18}
MDA	1.45	1.68	2.27		1		4.5<br>5.3	2.45×10^{-9}
MEL	-1.48	-1.19	0.63		1		5.3	7.73×10^{-12}
MET	-3.34	-3.33	0.39	1			3.0<br>11.9	3.12×10^{-14}
MPSA	-4.28	-4.38	1.10			1	1.7	1.06×10^{-6}
NDSA	-5.38	-5.38	1.35			1	-0.3<br>1.1	4.69×10^{-15}
NSA	-2.68	-2.69	2.30			1	0.7	1.00×10^{-8}
NTf2	0.51	0.51	2.19		1		-	3.28×10^{-3}
OLM	0.37	-0.25	3.40			1	2.4<br>3.7<br>5.1	6.41×10^{-19}
OXI	-0.39	-0.39	1.01		0.8	0.2	2.0<br>8.1	1.50×10^{-12}
PF6	-	-	-			1	-	-
PFPrA	-2.22	-2.24	2.89			1	0.6	9.27×10^{-4}
PFPrS	-2.37	-2.37	2.64			1	-3.6	1.12×10^{-4}
PLC9	-0.14	-0.14	1.27	1			-	6.09×10^{-11}
PRI	0.61	0.61	1.59		1		11.8<br>13.5	7.93×10^{-9}
PYC2	-3.24	-3.24	-0.61	1			-	8.16×10^{-9}
PYC3	-3.01	-3.01	-0.54	1			-	1.08×10^{-8}
SAC	-1.30	-1.30	1.87			1	-0.4<br>1.7	5.03×10^{-8}
SIP	-3.02	-3.02	2.19			1	0.2	4.27×10^{-12}
SMX	0.56	-0.55	1.86			1	1.8<br>5.7	3.91×10^{-11}
TCEP	1.42	1.42	1.64		1		-	1.04×10^{-6}
TFA	-3.09	-3.09	2.05			1	0.6	1.76×10^{-4}
TFMSA	-3.88	-3.88	1.18			1	-2.8	4.05×10^{-6}
THEO	0.13	0.10	1.28		1		8.7	6.86×10^{-11}
TPA	-0.08	0.53	3.15	1			10.7	8.17×10^{-3}
TPPO	3.67	3.67	2.94		1		-	2.15×10^{-8}
VEN	0.16	1.43	2.96	0.9	0.1		8.4<br>15.1	8.35×10^{-10}
VSA	0.62	-0.90	2.94			1	3.9<br>5.0	3.86×10^{-12}
XSA	-3.03	-3.03	2.13			1	0.7	1.25×10^{-7}

[b)] calculation with ACD/Percepta (v 2020.1.2, GALAS model)

[c)] dimensionless Henry's Law constant, data used from EPI Suite (EPIWEB v 4.1, HenryWin v 3.20, Bond Estimation model)[3]

Table S3. Method validation parameters for plant material and soil.

Abbreviation	linear range for plant material	R^2 for plant material	method detection limit in µg L^{-1}		apparent recovery [-]		
			leaves	roots	leaves	roots	water
AAMPS	0.5 - 10	0.990	0.18	0.18	0.5	0.5 ± 0.4	0.8 ± 0.1
AC22	0.05 - 10	0.965	0.01	1.46	1.2 ± 0	0.4 ± 0.1	1.0 ± 0.1
AC5	0.05 - 10	0.996	0.33	0.14	1.2 ± 0.2	1.1 ± 0.1	1.2 ± 0.1
ACE	0.5 - 10	1.000	0.25	0.02	0.7 ± 0.2	1.0 ± 0.1	1.0 ± 0.0
AG	0.5 - 10	0.997	1.76	0.52	1.1 ± 0	1.0 ± 0.2	1.1 ± 0
AHP	1 - 10	0.943	3.46	3.47	-	-	1.1 ± 0.1
AMB	0.5 - 10	0.994	0.16	0.24	-	-	1.3 ± 0.1
AOPSO3	1 - 10	0.957	1.93	4.35	0.6 ± 0	0.4 ± 0.1	0.9 ± 0
ATA	0.05 - 10	0.992	0.76	0.59	-	-	1.0 ± 0.1
ATY	0.01 - 10	0.994	1.7×10^{-3}	0.01	1.2 ± 0.1	1.1 ± 0	0.9 ± 0
BETMAC	0.1 - 10	0.999	0.06	0.01	1.5 ± 0.8	1.2 ± 0.4	1.1 ± 0.1
BF4	5 - 10	-	0.48	1.79	-	-	0.9 ± 0
BTZ	0.5 - 10	0.996	0.21	0.10	1.1 ± 0.1	1.5 ± 0.4	0.9 ± 0
CBZ	0.05 - 10	0.995	0.19	0.03	0.7 ± 0.1	1.2 ± 0.1	0.9 ± 0
CG	0.05 - 10	0.993	1.26	0.06	0.7 ± 0.1	0.7 ± 0.1	1.0 ± 0.1
CLIM	1 - 10	0.999	0.88	1.15	1.1 ± 0.1	0.9 ± 0	0.9 ± 0
CSA	1 - 10	0.991	1.10	0.86	0.6 ± 0.1	1.0 ± 0.3	0.9 ± 0.1
DBP	0.5 - 10	0.970	0.07	0.40	0.7 ± 0.1	1.2 ± 0.4	1.0 ± 0.1
DCF	0.5 - 10	0.985	0.29	0.07	1.3	1.8 ± 0	0.8 ± 0.1
DCHSS	0.5 - 10	0.987	0.02	0.11	0.7 ± 0.2	0.5 ± 0	0.6 ± 0.1
DIOTOG	0.1 - 10	0.996	0.17	0.03	1.0 ± 0.2	0.8 ± 0.1	0.7 ± 0.1
DMAEE	0.5 - 10	0.995	0.04	0.02	1.0 ± 0.2	1.1 ± 0	1.0 ± 0
DMDE	0.5 - 10	0.989	0.55	1.08	1.7 ± 0.2	0.5 ± 0	2.1 ± 0.3
DPG	0.01 - 10	0.998	0.92	0.69	1.0 ± 0.3	1.1 ± 0.2	0.9 ± 0
DTBSA	0.1 - 10	0.999	0.59	0.24	0.5 ± 0.1	0.7 ± 0	1.0 ± 0
DZA	0.5 - 10	0.996	0.43	0.16	1.0 ± 0.3	0.9 ± 0.2	1.1 ± 0.1
εCL	0.5 - 10	0.953	4.75	1.72	1.4 ± 0	1.7 ± 0.8	1.2 ± 0.2
GALA	0.1 - 10	0.990	0.07	0.07	1.2 ± 0.2	1.1 ± 0	0.9 ± 0.2
GUA	0.5 - 10	0.999	2.11	0.11	-	-	1.1 ± 0
HTMPE	0.05 - 10	0.999	0.95	1.28	1.5 ± 0.1	1.2 ± 0.1	1.0 ± 0
LOS	0.05 - 10	0.996	0.01	0.33	0.6 ± 0.1	0.7 ± 0.2	1.1 ± 0
MAPMA	0.05 - 10	0.999	0.01	0.17	1.1 ± 0.2	1.0 ± 0.1	1.0 ± 0.1
MAPTAC	0.1 - 10	0.998	0.12	0.18	1.0 ± 0.1	0.9 ± 0	1.1 ± 0.1
MDA	0.5 - 10	0.997	0.19	0.12	-	-	0.9 ± 0.1

MEL	0.05 - 10	1.000	0.50	0.39	1.1 ± 0.4	-	1.5 ± 0
MET	0.1 - 10	0.998	0.28	0.35	1.0 ± 0.2	0.9 ± 0.3	1.1 ± 0
MPSA	0.5 - 10	0.971	0.34	0.12	-	-	0.7 ± 0.1
NDSA	1 - 10	0.999	5.63	2.67	1.3	1.4 ± 0	1.1 ± 0
NSA	0.5 - 10	0.999	0.06	0.51	1.6 ± 0.8	0.6 ± 0.1	1.0 ± 0.2
NTf2	0.1 - 10	0.999	0.05	0.02	-	1.2 ± 0	1.1 ± 0.1
OLM	0.5 - 10	0.997	0.12	0.05	0.6 ± 0.2	0.9 ± 0.1	1.1 ± 0
OXI	0.5 - 10	0.993	1.30	0.26	0.3 ± 0	0.2 ± 0	1.0 ± 0
PF6	0.5 - 10	0.993	1.42	0.77	1.4 ± 0	0.8 ± 0	1.1 ± 0.1
PFPrA	0.1 - 10	0.998	0.25	0.20	-	1.5 ± 0	0.9 ± 0
PFPrS	0.5 - 10	0.994	0.06	0.22	1.2 ± 0.2	1.3 ± 0.1	0.9 ± 0.2
PLC9	0.05 - 10	0.999	0.15	0.04	0.9 ± 0.1	0.9 ± 0	1.1 ± 0.1
PRI	0.1 - 10	0.998	0.08	0.17	1.4 ± 0	0.8 ± 0	0.9 ± 0
PYC2	0.01 - 10	0.997	0.08	0.19	1.2 ± 0.2	0.8 ± 0.2	1.2 ± 0.1
PYC3	0.1 - 10	0.996	0.11	0.05	0.9 ± 0.2	0.7 ± 0.2	1.2 ± 0.1
SAC	1 - 10	0.998	2.60	0.37	-	-	1.4 ± 0.1
SIP	0.5 - 10	0.971	0.49	1.41	0.9 ± 0.2	0.8 ± 0	1.0 ± 0.3
SMX	0.1 - 10	0.996	0.09	0.84	1.0 ± 0.2	1.2 ± 0.1	1.0 ± 0
TCEP	0.05 - 10	0.999	0.66	1.77	1.0 ± 0.4	0.8 ± 0.2	0.9 ± 0.1
TFA	0.1 - 10	0.980	0.03	6.17	-	1.1 ± 0.2	1.4 ± 0.5
TFMSA	0.5 - 10	0.997	3.73	0.03	0.9 ± 0	0.8 ± 0	0.7 ± 0.2
THEO	0.1 - 10	0.998	0.24	0.43	0.7 ± 0.1	0.5 ± 0	0.8 ± 0.1
TPA	0.01 - 10	0.997	0.30	0.10	-	-	0.9 ± 0
TPPO	0.5 - 10	0.998	1.49	2.17	1.0 ± 0.3	0.9 ± 0.1	1.1 ± 0.1
VEN	0.05 - 10	0.999	0.01	0.02	1.1 ± 0.1	1.0 ± 0	1.1 ± 0.1
VSA	0.5 - 10	1.000	0.56	0.40	0.9 ± 0.1	1.1 ± 0.1	1.2 ± 0
XSA	0.5 - 10	0.997	0.01	0.06	1.4 ± 0	0.7 ± 0	1.0 ± 0

Results & Discussion

Table S4. Concentration of PM chemicals in rocket leaves and roots in the spike experiment (10 µg L^{-1}).

Abbreviation	mean c in rocket leaves in ng g^{-1} d.w.	relative sd of c in rocket leaves in %	mean c in rocket roots in ng g^{-1} d.w.	relative sd of c in rocket roots in %
AOPSO3	< LOD	-	< LOD	-
PFPrS	< LOD	-	< LOD	-
SAC	< LOD	-	< LOD	-
MDA	< LOD	-	< LOD	-
LOS	0.1	20.8	2.5	44.2
VSA	0.1	24.7	11.1	87.2
DCF	0.2	49.8	< LOD	-
HTMPE	0.2	33.3	< LOD	-
DTBSA	0.3	12.3	2.9	69.6
DCHSS	0.3	14.3	0.8	24.4
CLIM	0.3	21.9	0.7	11.8
BZT	0.4	22.9	0.9	14.6
THEO	0.6	15.7	1.7	24.0
GALA	0.7	18.1	0.3	65.4
SMX	0.7	8.9	1.9	33.3
MAPTAC	0.8	17.1	0.8	77.6
OLM	0.9	5.7	13.2	78.4
SIP	0.9	12.0	< LOD	-
DMDE	1.0	5.1	0.5	26.5
CSA	1.0	35.2	< LOD	-
PLC9	1.1	12.0	0.8	67.6
DMAEE	2.3	15.3	0.3	31.8
DIOTOG	2.4	10.0	2.2	64.7
ATY	2.5	49.8	0.6	66.6
DPG	2.9	27.9	0.2	52.5
AC22	2.9	12.6	2.9	44.7
DBP	3.6	26.4	2.3	10.3
MAPMA	4.1	4.6	0.8	54.2
VEN	4.7	18.5	0.4	52.1
eCL	5.0	7.7	6.1	35.4
MPSA	7.3	33.3	4.0	10.3
XSA	11.1	25.1	50.0	76.1
PYC5	11.1	5.9	16.5	36.1
NSA	12.3	26.0	14.8	0.6
PYC3	12.3	7.2	17.5	21.1
AC5	12.7	29.1	14.0	85.7
AMB	15.0	17.4	29.7	74.5
NDSA	15.7	27.6	29.2	78.6
TPA	20.9	45.5	1.9	18.5
GUA	21.6	35.6	9.4	14.1
BETMAC	26.5	25.0	39.5	64.2
AHP	30.3	26.2	40.3	72.3
MEL	34.0	23.5	15.5	61.8
DZA	35.3	19.7	24.9	79.7
ACE	41.2	31.0	7.7	80.6
TPPO	45.2	11.9	6.2	85.5
MET	45.5	21.4	42.9	70.6
CG	67.8	41.2	1.3	14.9
AAMPS	69.1	19.0	30.3	73.7
ATA	88.1	12.4	21.1	60.8
AG	132.4	25.5	1.3	24.5
TCEP	161.8	30.6	3.4	13.9

PF6	161.9	5.8	2.4	72.1
NTf2	171.8	11.8	24.9	76.2
CBZ	176.7	13.5	5.0	78.2
PRI	189.4	18.6	9.1	75.9
TFA	244.4	14.3	6.0	12.4
BF4	294.9	20.4	9.8	60.6
OXI	320.7	9.6	95.8	0.0
TFMSA	373.2	14.6	14.7	54.8
PFPrA	1106.0	6.9	27.7	73.5

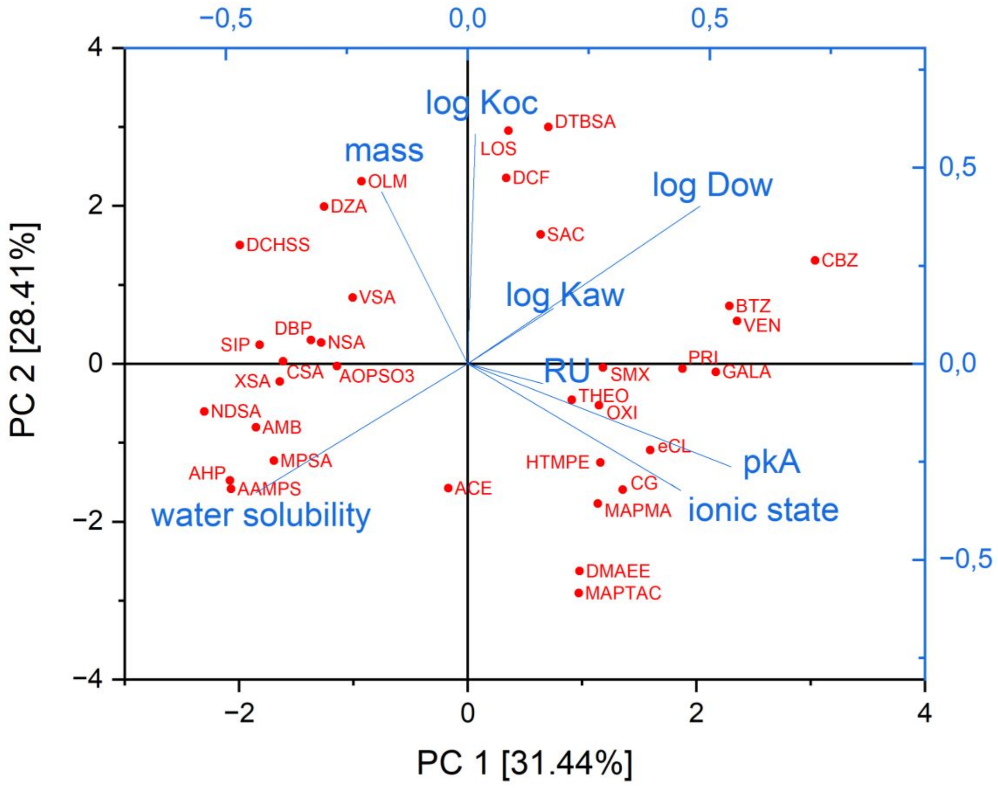

Figure S1. Principal component analysis of relative uptake (RU) values of spike experiment (10 µg L^{-1}) and physico-chemical properties.

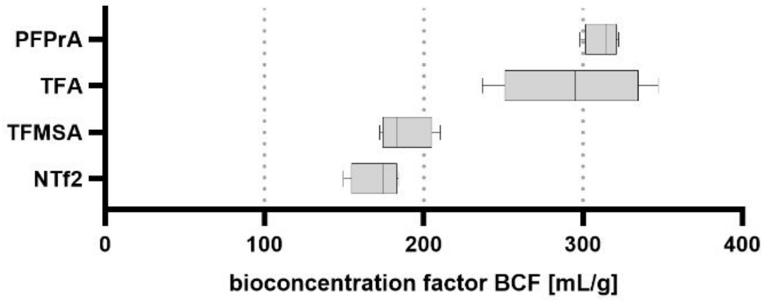

Figure S2. Bioconcentration factors of ultrashort-chain PFAS.

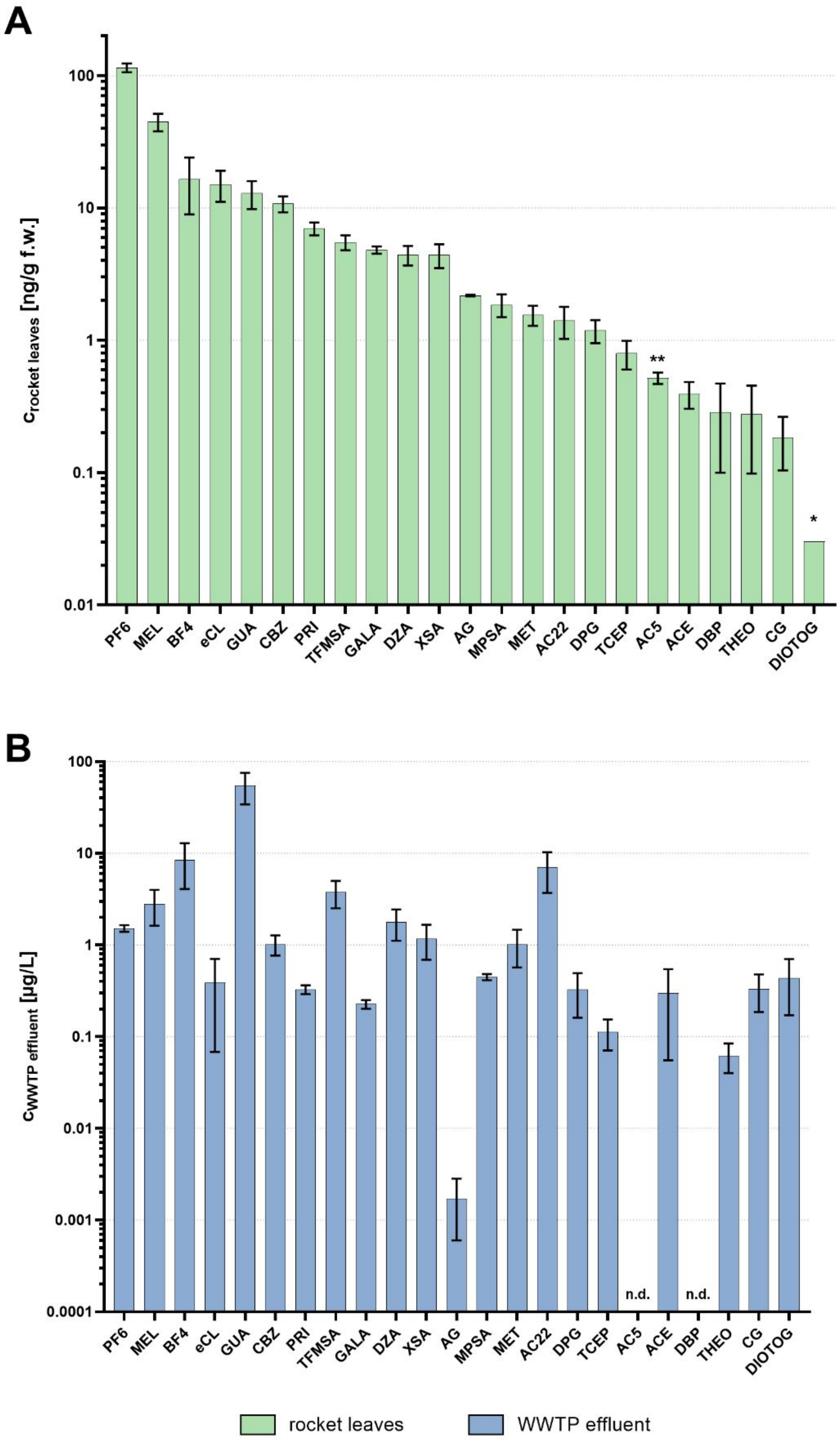

n.d. not detected
* only found in one replicate
** only found in two replicates

Figure S3. Concentrations of detected PM chemicals. a) Concentration in edible part of rocket in ng g^{-1} dry weight (d.w.) irrigated with WWTP effluent. b) Concentration in irrigation water (WWTP effluent) in µg L^{-1}, n.d. – not detected above limit of quantification.

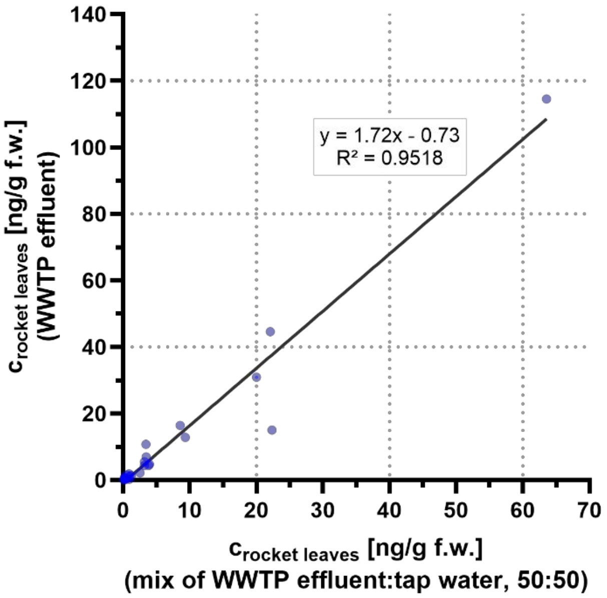

Figure S4. Dependence on concentration of irrigation water. Scatter plot of the amount in plant leaves irrigated with WWTP effluent and a mixture of WWTP effluent and deionized tap water (50:50, *v:v*).

Table S5. Maximum edible amount of rocket without exceeding the threshold of toxicological concern (TTC). Cramer Classes applied by using Toxtree software[4]. No Cramer Classes given for PF6, BF4, GALA and AC5. Criteria for TTC values used from EFSA[5].

Compound	Revised Cramer Class[a]	TTC level [ng/bw*day]	TTC value [µg/person[b]*d]	m_{max} [kg/person[b]*d]
MEL	3	1500	105	2.3
GUA	3	1500	105	8.2
CBZ	3	1500	105	9.8
TFMSA	3	1500	105	19
DZA	3	1500	105	24
XSA	3	1500	105	24
εCL	2	9000	630	42
AG	3	1500	105	48
MET	3	1500	105	68
AC22	3	1500	105	75
DPG	3	1500	105	89
PRI	2	9000	630	90
TCEP	3	1500	105	132
ACE	3	1500	105	266
DBP	3	1500	105	368
THEO	3	1500	105	379
CG	3	1500	105	571
MPSA	1	30000	2,100	1131
DIOTOG	3	1500	105	3474

[a] applied using Toxtree software[4]

[b] default value of 70 kg bodyweight (bw)

References

(1) European Chemicals Agency (ECHA). *Search for chemicals*. https://echa.europa.eu/information-on-chemicals (accessed 08.04.2025).

(2) Wishart, D. S.; Feunang, Y. D.; Guo, A. C.; Lo, E. J.; Marcu, A.; Grant, J. R.; Sajed, T.; Johnson, D.; Li, C.; Sayeeda, Z.; Assempour, N.; Iynkkaran, I.; Liu, Y.; Maciejewski, A.; Gale, N.; Wilson, A.; Chin, L.; Cummings, R.; Le, D.; Pon, A.; Knox, C.; Wilson, W. DrugBank 5.0: a major update to the DrugBank database for 2018. *Nucleic Acids Res* **2018**, *46* (D1), D1074-D1082. DOI: 10.1093/nar/gkx1037 From NLM Medline.

(3) U.S. Environmental Protection Agency (U.S. EPA). Estimation Programs Interface Suite™ for Microsoft® Windows, v 4.11. 2025. United States Environmental Protection Agency, Washington, DC, USA.

(4) Patlewicz, G.; Jeliazkova, N.; Safford, R. J.; Worth, A. P.; Aleksiev, B. An evaluation of the implementation of the Cramer classification scheme in the Toxtree software. *SAR and QSAR in Environmental Research* **2008**, *19* (5-6), 495-524. DOI: 10.1080/10629360802083871.

(5) EFSA Scientific Committee; More, S. J.; Bampidis, V.; Benford, D.; Bragard, C.; Halldorsson, T. I.; Hernandez-Jerez, A. F.; Hougaard Bennekou, S.; Koutsoumanis, K. P.; Machera, K.; Naegeli, H.; Nielsen, S. S.; Schlatter, J. R.; Schrenk, D.; Silano, V.; Turck, D.; Younes, M.; Gundert-Remy, U.; Kass, G. E. N.; Kleiner, J.; Rossi, A. M.; Serafimova, R. Reilly, L.; Wallace, H. M. Guidance on the use of the Threshold of Toxicological Concern approach in food safety assessment. *EFSA J* **2019**, *17* (6), e05708. DOI: 10.2903/j.efsa.2019.5708 From NLM PubMed-not-MEDLINE.
